# ‘The System is Not Set up for the Benefit of Women’: Women’s Experiences of Decision-Making During Pregnancy and Birth in Ireland

**DOI:** 10.1177/10497323211055461

**Published:** 2021-12-01

**Authors:** Susann Huschke

**Affiliations:** 1 Public and Patient Involvement (PPI) Research Unit (School of Medicine) and Health Research Institute, 8808 University of Limerick, Limerick, Ireland

**Keywords:** childbirth, reproduction, decision making, power, empowerment, feminism, gender, pregnancy, doctor-patient, nurse-patient, communication, informed choice autonomy

## Abstract

In this article, I draw on in-depth qualitative interviews with 23 women, conducted in 2019/2020, focusing on their involvement in decision-making during pregnancy and birth. The study is located in Ireland, where comparably progressive national policies regarding informed choice in labour and birth clash with the day-to-day reality of a heavily medicalised, paternalistic maternity care system. I represent the subjective experiences of a diverse group of women through in-depth interview excerpts. In my analysis, I move beyond describing *what* is happening in the Irish maternity system to discussing *why* this is happening – relating the findings of the research to the international literature on authoritative knowledge, technocratic hospital cultures and risk-based discourses around birth. In the last section of the article, I offer concrete, empirically grounded and innovative recommendations how to enhance women’s involvement in decision-making.

## Introduction

In this article, I discuss the experiences of a diverse sample of women in Ireland with regard to their involvement in decision-making during pregnancy and birth. The analysis offered in this article is grounded in feminist approaches to reproductive health, where a women’s right to make informed choices regarding their care is viewed as an essential, fundamental aspect of women’s liberation (e.g. Apfel, 2016; Hill, 2019; The Boston Women’s Health Book Collective, 2008; Murphy-Lawless, 1998; Ehrenreich & English, 2009; Wickham, 2018).

The legal basis for pregnant people’s^1^ right to make decisions regarding medical tests, treatments and interventions during pregnancy and birth is established in common law throughout Europe, supported by international declarations (e.g. WHO, 1994) and national policies Health Service Executive (HSE, 2019). However, despite the clear legal grounds for informed decision-making, the international research literature demonstrates that these rights are not always upheld in the everyday interactions between pregnant people and maternity care providers. Women report feeling disrespected and ignored during labour and birth in maternity care settings where they are treated as objects rather than active subjects by medical professionals (Nilsson, 2014; Ijadunola et al., 2019; Vedam et al., 2019). In order to ensure women’s compliance and discourage autonomous decision-making, women are frequently only given insufficient or biased information by their health care providers (Jay, 2015; Jenkinson et al., 2017), combined with ‘emotional blackmail’ (Kitzinger, 2006) used to ensure their ‘consent’ to the option preferred by the medical professional (O’Hare & Fallon, 2011). A growing body of research, inspired by childbirth activism in Latin America, describes these interactions as forms of *obstetric violence*, which also include non-consensual vaginal examinations (Burrowes et al., 2017), unwanted episiotomies (Babiaková et al., 2015) and forced C-sections (Diaz-Tello, 2016).

These common practices may often be an unintended result of the working conditions in maternity systems that are under-resourced and under pressure to be economically efficient. Clearly, however, the disregard of birthing people as active subjects with the right to make decisions regarding their bodies, their babies and their births has significant negative effects on their wellbeing: ‘decreasing autonomy risks decreasing women’s sense of self-worth, self-trust, self-esteem and confidence’ (Edwards, 2004, p. 20). Research has shown that a lack of involvement in decision-making and a sense of being powerless and out of control can result in traumatic birth experiences (Creedy et al., 2000; Soet et al., 2003). The effects of negative birth experiences include lower quality of life postpartum (Smarandache et al., 2015), lower self-rated health (Schytt & Waldenström, 2007) and symptoms of post-traumatic stress disorder (Garthus-Niegel et al., 2013, 2014).

At the same time, research has shown that a positive birth experience can ‘enhance a woman’s sense of self-esteem and emotional well-being’ (Thachuk, 2007, p. 53). In midwifery models of care and in the reproductive justice and birth rights movements, the ‘process of pregnancy and childbirth [is understood] as a potentially momentous psychological, social, and spiritual events in a woman’s life’ (Thachuk, 2007, p. 46). That is to say, birth matters (cf. Gaskin, 2011) and respecting the birthing person’s autonomy and their role in informed decision-making is one of the basic principles that need to be in place in order to facilitate a positive birth experience.

This article contributes to the field of critical feminist research on pregnancy and birth by representing the subjective experiences of a diverse group of women through in-depth interview excerpts. In my analysis, I move beyond describing *what* is happening in the Irish maternity system to discussing *why* this is happening – relating the findings of the research to the international literature on authoritative knowledge, technocratic hospital cultures and risk-based discourses around birth. In the last section of the article, I offer concrete, empirically grounded and innovative recommendations how to enhance women’s involvement in decision-making.

### Background: Giving Birth in Ireland

The right to make informed choices in healthcare is formally established in Ireland: the Irish Health Service Executive (HSE) National Consent Policy (2019) very clearly acknowledges ‘the service user’s right to self-determination (or autonomy) – their right to control their own life and to decide what happens to their own body’ (HSE, 2019, p. 20). The policy addresses the potential conflict between health care providers, who ‘can often claim greater expertise in decisions regarding the “means” to achieve the “end” of better health’, but asserts that service users retain ultimate decision-making authority:

Service users are the experts in determining what ‘ends’ matter to them, including how they should live their everyday lives, decisions about risk-taking and preference for privacy or non-interference (HSE, 2019, p. 21).

In order for consent to be valid, service users need to have received ‘sufficient information in a manner that is comprehensible to them about the nature, purpose, benefits and risks of an intervention’ (HSE, 2019, p. 23). Furthermore, consent needs to be voluntary, that is, service users need to understand that they have a choice, and the ‘use of threats to induce consent (…) is not acceptable’ (HSE, 2019, p. 29).

These clear national guidelines jar with the reality of the Irish maternity system, which has long been criticized as paternalistic and hierarchical, embedded in a culture of distrust in women (Murphy-Lawless, 1998). The vast majority of women give birth in a hospital (HPO, 2018). Midwifery has been marginalized and underdeveloped (Kennedy, 2010, 2012). There are no stand-alone midwifery-led units in Ireland, and homebirth services are not accessible to many women. C-section rates are high at 32.7% – significantly higher than the 10–15% that the World Health Organisation recommends as the ideal rate to improve health outcomes (WHO, 2015). In the public maternity system, women usually see different obstetricians and midwives throughout their pregnancy with no continuity of carer. Women who can afford it may opt for private health insurance and/or out of pocket payments for private care, creating a two-tier system.

The dominant beliefs about childbirth and the professional and organisational culture of the Irish maternity system can be summarised as ‘technocratic obstetrics’, as described by Davis-Floyd (2001). In the technocratic understanding of birth, the female body is viewed, essentially, as a defective machine, an object, which requires obstetric interventions to ‘conform to the assembly-line model of factory production’ (Davis-Floyd, 2001, S6). Technocratic hospital cultures are highly hierarchical and subordinate women’s ‘individual needs to standardized institutional practices and routines’ (ibid). In the labour ward of one of the biggest maternity hospitals in the country, a poster reads in large letters ‘It is not necessary for you to write a birth plan.’

While this can be read as an expression of concern for pregnant people’s wellbeing and the acknowledgment that birth often does not go ‘according to plan’, it does leave the impression that overall, the public maternity system does not encourage birthing people to make their own autonomous decisions (cf. AIMS, 2014). The shortcomings of the technocratic Irish maternity system are well documented. Several studies have highlighted that a proportion of women are dissatisfied with their involvement in decision-making and the lack of choices available to them (AIMS, 2014; NCEP, 2020; Larkin et al., 2017). A small number of qualitative studies on decision-making in the birth space have highlighted the importance of the social context of decision-making, in particular the relationship between the birthing person and their care provider (O’Brien et al., 2018; O’Hare & Fallon, 2011).

Building on these studies, this article adds new insights by exploring how the structure of the maternity system affects the concrete relationships and interactions between birthing people and their care providers. Drawing on in-depth qualitative case studies, I offer recommendations for macro and micro level changes that are needed in order to expand women’s involvement in decision-making.

## Methodology

The aim of this study was to investigate women’s subjective experiences of mental health and wellbeing during pregnancy, birth and postpartum. While situated in the wider field of research on perinatal mental health, this study is grounded in critical medical anthropology and draws on feminist research methodologies. In critical medical anthropology, health and illness are explored as *subjective experiences* through ethnographic methods, including extended interviews, with the aim to understand how the lives of individuals are affected by large-scale social, political and economic structures, including hospital cultures and medical authority. Mental health, understood in this way, is always an *emic* category: it is subjective and context-specific, and needs to be defined from within a person’s social and moral world (cf. Kleinman & Kleinman, 1991; Biehl, 2005). Furthermore, the presentation of findings in this article connects to the long-standing tradition in feminist research to use personal narratives and case studies to shed light on the effects of social structures and dominant cultural norms on the lived experiences of people (e.g. Abu-Lughod, 2008).

This article draws on in-depth qualitative interviews with 23 women in Ireland who were either pregnant at the time of the interview (*n* = 4) or had given birth in Ireland in the past 12 months (*n* = 19). Pregnant participants were interviewed again after the birth to include their most recent birth experience. The interviews were conducted between March 2019 and February 2020 by the author. The study received ethics approval by the Faculty of Education and Health Sciences Research Ethics Committee of the University of Limerick in February 2019.

**Figure 1.**
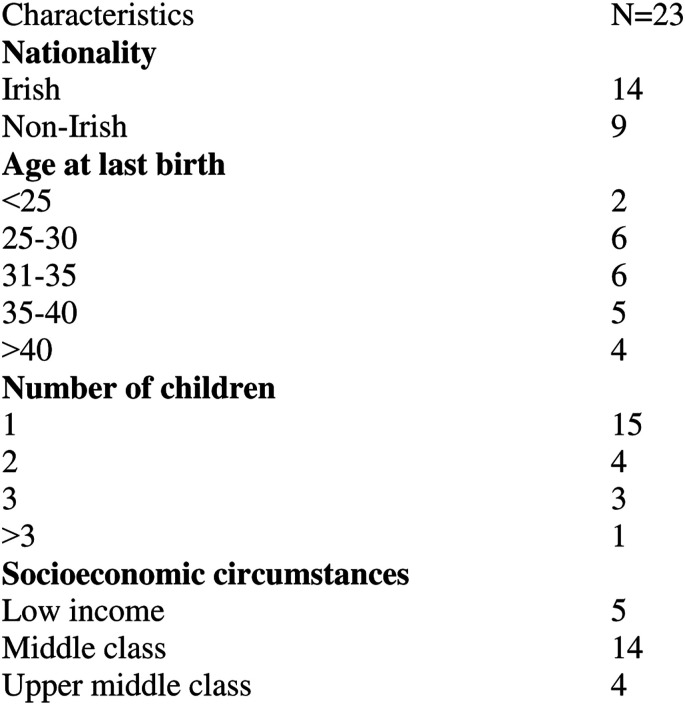
Characteristics of the study participants.

The study employed purposive sampling with the aim of interviewing a diverse group of women. Participants were recruited via personal networks, online forums, voluntary sector organisations, and via snowballing (i.e. participants inviting others to take part). Participants were interviewed in different locations throughout the Midwest region. Their births took place in different hospitals across Ireland. Of the 23 women interviewed, 14 were Irish and 9 were non-Irish citizens. Their age ranged from 25 to 47 years. Thirteen of them were first time mothers. Most of the women described their socioeconomic circumstances as middle class, with four women identifying as upper middle class. Five women described their socioeconomic circumstances as difficult, including having low income, living in refugee accommodation or being homeless (Figure 1).

In line with the study methodology focusing on subjectivity and lived experience, the interview questions were open-ended and aimed to solicit narratives that focused on how the interviewee *felt* throughout the various stages of the perinatal period. All women talked about their interactions with health care providers during their pregnancies and shared their birth stories. The (lack of) involvement in decision-making constituted a dominant theme in the narratives, and interviewees felt that it significantly shaped their mental health and wellbeing. Follow-up questions were asked in the interviews to solicit detailed descriptions of the decision-making process during labour and birth, and to understand the woman’s feelings about this.

During (manual) inductive thematic analysis of the interview transcripts (cf. Braun & Clarke, 2006; Nowell et al., 2017), decision-making during pregnancy and birth was coded as one of the key themes. In regard to the importance of decision-making and autonomy, theoretical saturation (cf. (Bernard, 2011)) was reached with the 23 women interviewed, that is, the narratives shared by new interviewees confirmed existing analytical themes rather than adding new themes. Within the topic of decision-making, four subthemes emerged during the data analysis – three relating to negative experiences of decision-making, and one relating to positive experiences: (1) lack of information and involvement, (2) the struggle to practice informed refusal (3) organisational barriers, and (4) autonomy and person-centred care. These subthemes were used to structure the results section below.

### Ethics, Positionality and Reflexivity

All participants received an information sheet about the research prior to the interview via email. They had the opportunity to ask questions about the study and the researcher’s background, and signed an informed consent sheet before the recording of the interview. In addition to this, I ensured on-going consent by reiterating throughout the interview, particularly when the conversation caused deep feelings, that the interviewee did not need to talk about experiences that were too painful to share. This process requires sensitivity and empathy and cannot be formalised. As a researcher, activist and birth doula, I have extensive experience of engaging with people who have experienced trauma and/or are currently experiencing emotional distress (e.g. Huschke, 2015; 2017a; 2017b). In this study, all participants expressed at the end of the interviews that they were glad they had talked about their experiences, including painful and upsetting aspects, and felt that being listened to was a positive experience. I made sure that each interview ended on a positive note, to ensure I did not leave people feeling upset or emotionally drained. All interviewees were given a handout with counselling and support services. The names of all participants were changed to protect their anonymity.

As with any research project, this study is informed by my interests, my professional background and my personal experience. The aim of rendering my background and interests visible in the following paragraphs is to increase transparency and integrity in this piece of research, in line with feminist research methodologies (e.g. Haraway, 1988; Davis & Craven, 2016) and international best practice in qualitative health research (e.g. Lambert et al., 2010; Doyle, 2012; Krusz et al., 2020; Bourke, 2014). I am a medical anthropologist by training, and all my research projects have adopted a critical lens, exploring issues of autonomy, choice and in/equality in the context of interactions with health care providers. As a feminist, a mother of two, and a birth doula, I am not dispassionate about the issues discussed in this article. On the contrary: my work, both as a researcher and a doula, directly aims to increase equality and equity within the Irish maternity system, create more choices and ensure respect for the autonomy of birthing people (Figure 2).

**Image 1.**
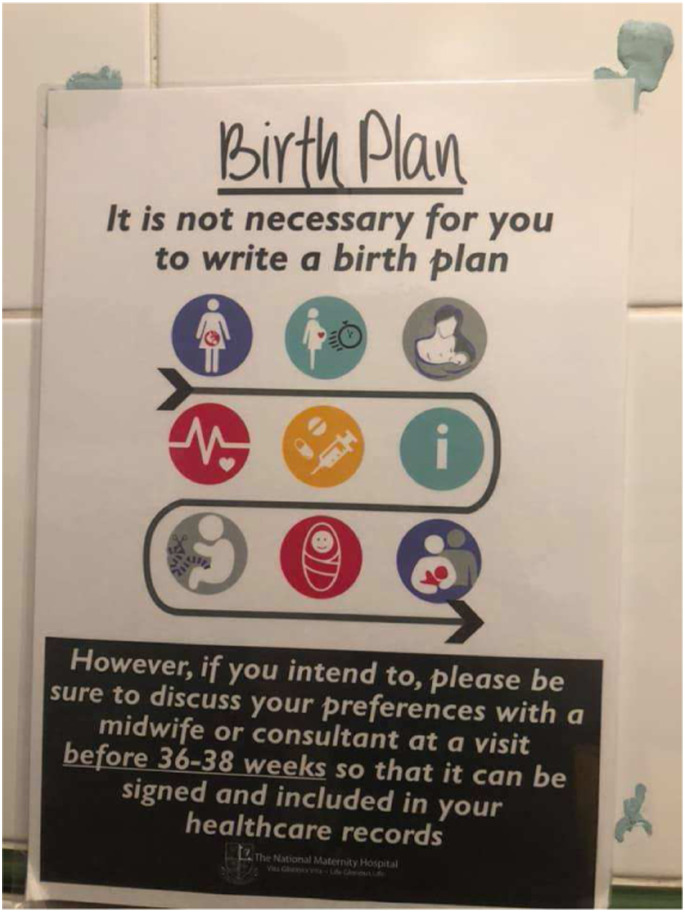
Poster in an Irish labour ward (2019).

My background constituted a strength of the research: my experience and knowledge facilitated the recruitment of participants, allowed me to relate to women’s stories, express empathy and ask relevant follow-up questions. It likely also contributed to the rapport I established with women in the interviews, allowing deep conversations to emerge (cf. (West & Bartkowski, 2019)). In order to make certain that the analysis presented in this article is valid, that is, that it represents the lived realities of the people it claims to represent, and reliable, that is, that the findings would be confirmed if more research was conducted or if someone else studied the same research question, I offer the following: firstly, the sample was designed specifically to move beyond the bias of talking to a homogenous group of women. It was also important that the invitation focused on the broad area of emotional wellbeing, rather than predetermining ‘decision-making’, ‘choice’ and ‘autonomy’ as topics, which could have led to a more selective sample of women who have strong views on this topic. In this way, the findings presented in the article are grounded in the lived experiences of women from different backgrounds, with different hopes and expectations for their births. Secondly, the findings presented here are in line with the existing literature on decision-making in the birth space, both in Ireland and internationally – the lack of informed choice practices is a known problem in technocratic, medicalized maternity systems, as described in the introduction. Thirdly, I sent this article to two of the interviewees to ask for feedback, as well as to two birth professionals with long-standing experience of working in the Irish maternity system. These stakeholders felt that the findings correctly represented their lived experience. In regard to the conceptual lens and the conclusions drawn from the findings: these are, as in any study, shaped by my positionality, and reflect my interest in increasing relational autonomy in the birth space by making the subjective experiences of birthing people the focal point of my analysis. It is important, however, to differentiate between the validity and reliability of the findings themselves, and the subjective nature of the recommendations and conclusions.

In the following, I represent some of the experiences that women shared with me. The case examples and quotes were selected because they illustrate the respective theme most vividly, *and* with a view of including the experiences of different women – for example, Irish/non-Irish women, working class/middle class women, first time mothers/experienced mothers. Inspired by feminist writing traditions (cf. Davis & Craven, 2016, p. 22), I opted to present the themes through case stories rather than depersonalised quotes, as a way of rendering the research participants visible as unique people and situating their views on decision-making within the broader context of their expectations and lived experience.

### Women’s Stories: Study Findings

The following themes are overlapping, and the quotes often touch on multiple aspects at the same time. All names are pseudonyms.^2^

#### ‘Why didn’t anybody tell me?’ – Lack of information and involvement

The experiences shared by women in this study indicate that some of the interactions between the birthing person and their health care provider take place in a grey zone where consent is not explicitly given. They also describe circumstances in which women agreed to a certain course of action, but felt, in hindsight, that they did not have the information they needed to actually make this decision. These experiences thus highlight breaches in the legal requirement for *informed* consent for all interventions.

#### Sabina

Sabina is a Latvian mother of a toddler and a pre-schooler. Sabina had planned a homebirth for her first child, but ended up transferring to the hospital after many hours of labouring at home because her electricity was turned off due to maintenance work. She recalls her experience of birthing in the hospital:

They were waiting for dilation [the opening of the cervix], they were threatening with Caesarean which we didn’t want. We were praying, everybody was praying. No Caesarean, we wanna go natural. The hospital was a shocker for me because people coming in every few minutes to check on me. Everyone wanted to check me and I was a bit annoyed with that, like everybody wanted to kind of shove their fingers in there. I just wanted to rest, I got my epidural [pain relief] and I was just trying to sleep, and people were popping in all the time. (…) Finally I was open [fully dilated] according to one of the doctors. They wanted me to push and they started using the suction cup. But I think I would have pushed him out on my own because they took off epidural and I felt him going down, and if they gave me another extra 20 minutes or half an hour then he would have come out by himself, but they wanted him out. It was the middle of the night so they wanted him out.

In her telling of the story, Sabina describes how her needs and wishes were not fully taken into account in the hospital. The vaginal examinations were performed as a routine practice, without her explicit consent. Similarly, she was not given the opportunity to refuse the coached pushing and the use of a suction cup, despite the fact that she had opted for a homebirth in order to have an intervention-free birth. If there was a medical reason why she needed to push her baby out urgently, this was not explained to her, leaving her feeling coerced into coached pushing and the instrumental delivery via the suction cup.

#### Rachel

Rachel is an Irish mother of a 1 year old. Rachel experienced an easy pregnancy, followed by a difficult birth. When she went into the hospital for her routine 38-week check-up, her consultant performed a membrane sweep and broke the waters in the process:

Rachel: At thirty eight weeks they did a sweep [internal sweeping of the cervix to start labour] on me. And the doctor who did the sweep broke my waters.

Susann: Why did they do a sweep?

Rachel: Because… I don’t know why really. The doctor who did it was my consultant, I was a public patient. He was just doing the rounds and I kind of trusted him in a way, do you know, that the sweep wasn’t going to do any harm. But actually in looking back I wouldn’t do a sweep again, I wouldn’t let them, definitely not, because I was GBS [Group B Streptococcus] positive as well, so I knew that once my waters were going to go I had to be on an antibiotic drip. And had I known what would come out of it, that I had to be put on the [oxytocin] drip and stuff… I just would have let it happen as naturally as possible, and not let anybody near me.

Susann: Can you remember the conversation that you would have had with the consultant before he did the sweep?

Rachel: I just remember lying down, and he was like ok let’s have a look, and yes obviously check your cervix (…) and I guess he would have said that I was in a good position for a sweep and I would have just said, ok, but yes.

Susann: And did you know what a sweep was?

Rachel: Yes, I knew that they wipe the sac basically if they can see it, but I didn’t know that it would break my waters that instantly. So when I went up to the ward then that night like the nurses were saying, oh that doctor is well known for breaking peoples waters because he has long nails. Like oh Jesus guys, so yes, had I known that… See after that then everything kind of went in a waterfall like of what came next and next and next and I should have just said no, I will just let it happen as naturally as possible.

Rachel was admitted to the maternity ward. She was given prostaglandin gel to induce labour. Her partner Davie left late that evening because he was not allowed to stay overnight on the ward, so she was on her own when she was brought up to the labour ward at 2 am and put on an oxytocin drip to start uterine contractions.

I don’t think I understood that I would be put on an oxytocin drip [to induce labor] and I didn’t really know what to expect. I tell everyone now it was the worst thing in the world. Because of what my own experience to avoid it at all costs. [When Davie came back in the morning] I had been up all night then going through this drip by myself, and I was really anxious and nervous and scared I suppose. [Starts crying].

Rachel gave birth to her baby the next day, after an hour of coached pushing, an episiotomy [cutting of the perineum between vagina and anus] and the use of a suction cup. She sums up the birth:

It was hard like, yes, when I think back of the hours that I went through like on my own (…) there is a lot there that they put you through without realizing. All from a sweep. I will never let anybody go near me again you know. (…) You are kind of afraid because you know that the nurses and doctors are there to do that job, to deliver the babies, so if you say anything kind of out of the ordinary… you kind of you feel like you have to get on with it and that is it.

Rachel’s experience exemplifies the potential effects of a lack of information: she was not made aware of the risks involved in a membrane sweep, nor was she told what the benefits were in her specific case. In hindsight, she wishes she had known, and had been given the opportunity to refuse consent. Rachel’s reference to not wanting to say ‘anything out of the ordinary’ out of fear of challenging medical authority further relates to the experiences of women who *did* refuse certain procedures, and found the responses from medical professionals to be negative and stressful, as described in the next section.

#### “We have a troublemaker here”: informed refusal

Some of the women interviewed, recounted experiences where they refused an intervention or routine test in a maternity hospital. They felt that they were put under pressure to conform with the hospital protocol, leaving them feeling frustrated and disrespected.

#### Delia

Delia is a Brazilian–Irish mother of a small baby. Her decision-making was informed by her desire to have a physiological birth at home. She found an HSE homebirth midwife that cared for her during her pregnancy. However, because Delia went more than 14 days past the estimated due date (i.e. had a ‘post-date pregnancy’), she was ‘risked out’ of the HSE homebirth scheme and her care transferred to the maternity hospital. Delia insisted that the due date was calculated wrong and did not reflect her actual reproductive cycle. Delia offers a very vidid description of the decision-making process regarding an induction for post-dates, which exemplifies the threatening language used by some medical professionals to ‘encourage’ women into choosing ‘the right option’.

Delia: Because I was so long overdue I had to go every day for check-ups. My consultant saw me that Wednesday and he was very rude. He came to me, and my partner was there, and the consultant was very pissed off. He was like, why natural? Why do you have to go natural? You’re being irresponsible, there’ll be meconium in the water, your baby is going to be born dead, and all this blah blah blah to me.

Susann: He actually said that?

Delia: He said all that to me that day, so I was (…) really emotional, I was nearly crying. But I had the information with me. I know what I’m doing here even though it was very hard to fight against that. The consultant said to my partner that, if something goes wrong I know exactly what’s going to happen, you’re gonna sue us. So that was a bit annoying that he would see this insurance wise, medical negligence straight away, not even paying attention to what we’re trying to say. (…) I was 42+2 weeks and I said, I want to go to 43 weeks and then I decide what to do, and he said, no you can’t. I said, no I can. Then they did an ultrasound and the scan was showing a decrease on my [amniotic] fluid. So they tried to say, see, now you have a sign of something dangerous could happen, and I said, yeah but it’s also a sign that labor is approaching. They were quite hard to argue with. It was an awful experience. I managed to bribe my way out by scheduling an induction for Friday at 8am.

Delia went into labour the night before the scheduled induction, and was told the next morning in the hospital that ‘induction is officially off the table’. Her baby was born 2 days later in the hospital, at 42+5, without any major interventions. Delia was delighted with herself and the birth.

#### Mel

Mel is a US American woman. She has four children and is pregnant with the fifth. Mel has struggled with mental health issues in the past and is very aware of triggers for her anxiety. After two traumatic births, she passionately feels that intervention-free pregnancies and births are right for her. Mel describes her interactions with hospital staff when she went in for her booking appointment at 17 weeks in her current pregnancy. She did not want an ultrasound scan, which led to lengthy discussions with the staff, leaving her feeling stressed and upset.

Mel: I didn’t want a scan and that led to a whole kind of intervention, trying to convince me otherwise, you know. (…) I think because of my immigration status people talk to me like I don’t know the cultural norms of Ireland. So the midwife was like, well in Ireland and in this hospital this is what we do. And that was when they took me to another room, with the director of antenatal care, I don’t know, but she brought me in there and explained, you have to have a scan, every single woman who comes in here has to have a scan.

Susann: And how did they explain to you why you needed to have a scan?

Mel: They had no reason, their reason was this is what we do, this is our protocol. But I was asked by this director, you know, why don’t you want to do it? (…) I’m not comfortable with the level of sound waves, I’m not comfortable with heating up a fetal tissue. But I was also aggravated because I didn’t feel like I should have to explain my choice.

Susann: Especially if they’re not explaining their choice.

Mel: Yes. (…) I’d say a lot of women feel this way, it’s like, oh we have a troublemaker here, we have somebody that doesn’t want to follow our rules. And that’s kind of how I felt. And then the midwife pulled in a consultant, she was like, you know we have someone here who doesn’t want to have a scan. And then she asked the same thing, well why don’t you want to have one and I was like oh, I was just so frustrated, and I said the same thing to her. And then the consultant was the one who said well we can’t force her to have one if she doesn’t want to have one, which I already knew.

Susann: So that was the first time that got mentioned throughout this.

Mel: Yeah that was the first time informed refusal was actually even brought up in the conversation. (…) I left that day feeling like had been emotionally like beaten up. I had a headache before I even left. I was doing like deep breathing because I was anticipating every new person I met was going to ask the same fucking questions, why don’t you want to do this, this is our protocol.

Both Delia’s and Mel’s experience highlight how difficult it can be for women to make a choice that does not align with the hospital protocol. The stories presented in the following section further illustrate how the dominant organisational culture of Irish maternity hospitals does not facilitate woman-centred care and autonomous decision-making.

#### ‘The system is not set up for the benefit of women’: Organisational barriers

In their descriptions of their births, women often referred to hospital policies as reasons for the course of action that was taken. Some women felt that their personal needs, views and experiences were ignored in the decision-making process, and that decisions were made not for their benefit or the benefit of their baby, but for the benefit of the hospital or the care provider.

#### Alice

Alice is an Irish mother of three children aged between 4 years and 5 months. Alice experienced her interactions with the maternity system overall as difficult. She recounts a number of experiences over the course of her three pregnancies where she felt that she was not informed, supported or heard in the process of decision-making.

During labour with her second baby, Alice felt that decisions were made not for her or the baby’s benefit, but to make her fit into the obstetrician’s schedule:

There was a midwife there, I can remember it really clearly, and she was saying she needs to be pushing now, we need to get her pushing now. And the doctor strolled in at one point and he looked at the clock and said she can start pushing at 12 o’clock, I’ll never forget it, and he walked out of the room. And there was a senior midwife and this other midwife, and the midwife says we need to get this, we need to get her pushing, we need to get the baby born and the senior midwife says no, we need to listen to the doctor. I can remember, I talked to James [her partner] about it afterwards, I was like did that actually happen or was that in my own mind. He was like no, no, that totally happened. So yeah, so the doctor came back, I don’t know if he was having his lunch break or dealing with another woman or whatever. (…) It made me angry about the system and the way the system is and how unsupportive it is and how it’s really not set up for the benefit of women in any way shape or form, it’s completely set up for the benefit of the hospital and the staff and managing the hospital and managing their space. It just made me really mad.

#### Mandy

Mandy is an Irish mother of a primary school kid and a toddler. Mandy described her first birth as traumatic. She was diagnosed with gestational diabetes – although she feels that she was ‘borderline’ as she was managing it very well with her diet. A decision was made by the obstetric team to induce her at 38 weeks. In hindsight, Mandy feels that her voice was not heard in the decision-making process. Her husband works abroad and could not be present for the birth due to the early induction. She experienced a long, exhausting labour, which involved being induced with a pitocin (i.e. artificial oxytocin) drip, which was then stopped after a day because there was no space in the labour ward overnight. Instead, she was given a sedative to put her to sleep until the next morning, when there was space in the ward to continue her labour. Looking back, Mandy feels that this process contributed to the traumatic birth:

I was panicked, I was alone, I didn’t know any better. I suppose I was just trusting that they had our best interest at heart. But now I kind of feel like you wouldn’t treat an animal like that, vets would not do this to a cow who was in labor in the field. I just felt like I was treated like an animal who was an inconvenience.

After nearly 40 hours of labour, the midwife ruptured her membranes to speed up the process.

The baby wasn’t ready (…) and he got into trouble, his heartbeat started dropping, and he basically got stuck in the birth canal. They didn’t know whether he was in or out, you know, and he was really struggling.

Eventually, a consultant performed an episiotomy and her baby was born. Mandy feels that this traumatic birth experience could have been prevented:

It was something that was directly as a result of some administrative decision you know as in women with gestational diabetes have to have babies at thirty eight weeks you know that is a decision someone had made and in probably ninety per cent of cases it was fine. But then you marry it with a system that is overcrowded you know and that is where it comes a lack of care you know that is where it becomes a problem.

In addition to the difficult birth, Mandy experienced significant and long-lasting issues with the episiotomy, which did not heal well, rendering her postpartum experience traumatic. She was later diagnosed with postpartum depression.

#### ‘Somebody who understands where you’re coming from’ – Autonomy and person-centred care

In the following, I will present three positive experiences of decision-making during pregnancy and labour: a free birth, a hospital birth with a private midwife and an elective C-section. These stories illustrate some of the factors that need to be in place for women to feel heard, supported and respected as the primary decision-makers during their pregnancies and births.

#### Zoey

Zoey is an Irish mother of one. During the pregnancy with her firstborn baby, Zoey was apprehensive of giving birth in a hospital, due to her mother’s traumatic experiences when Zoey was born, and fears of unwanted interventions. She tried for several months to find an HSE homebirth midwife, but the only midwife covering her area was not available around her due date. Weighing her options, Zoey chose to have a free birth (a planned unassisted homebirth) instead of a hospital birth. She described the birth:

It was amazing. (…) I was on my knees because it was just the most comfortable for me. But I was able to push when I felt like I needed to and it wasn’t, there was nobody telling me like push now, you know push now. It was really, really good. I felt really powerful.

Zoey knew that an intervention-free, undisturbed birth was right for her, and she felt that she would not be able to have that in the hospital. She felt very positive about her birth experience, but would have still preferred to have a midwife present. Zoey was pregnant again the time of the interview. For her second baby, she was again not able to access an HSE homebirth midwife, but hired a private midwife to care for her.

#### Alice

Alice, who had experienced aspects of her first and second birth as difficult, hired a private midwife for her third birth to facilitate a homebirth. During labour, Alice was transferred to the hospital because her waters had broken, and labour had not started, which was a concern because she had tested positive for Group B Strep. Alice feels strongly that the presence of her private midwife changed the way the hospital staff interacted with her and helped ensure that her views and preferences were respected:

We went into the hospital, they were quite good in the hospital, they knew that I’d planned a home birth and that’s what I wanted. It made a big difference having Maura [her midwife] there, just her presence. Suddenly everyone is on their best behavior. Suddenly everybody is watching their Ps and Qs,

During the second stage of labour, Alice felt that the presence of her midwife prevented her giving birth on her back, a position she was not comfortable in:

Maura walked into the room then and said get her up on her knees. So she [her third baby] would have been born, again, with me on my back I’d say had it not been for Maura, because I just wasn’t, you know, *there* enough to kind of say no I want to be on my knees. (…) I would say things would have gone differently without her. Even though I was more educated, even though I knew what I wanted, even though you know I felt very strongly about what was wrong and what was right and how I wanted things to be. I still needed her there. She was a huge support (…), somebody who understands where you’re coming from.

#### Mandy

After the traumatic birth and postpartum experience with her first child, Mandy paid for private care for her second pregnancy, with the same consultant that had delivered her first child. She felt that this significantly improved the care she received: she was involved in the decisions, and they were made with her best interest at heart. She experienced a lot of complications during that pregnancy, and taking her experience of birth trauma, postpartum depression and anxiety into account, she agreed that an elective C-section would be the best option for her:

I felt relief, I will be honest, because it was something that could be planned for I thought, and it was something that could be approached logically, systematically and I knew my obstetrician would be in the room you know.

She appreciated the effort that the midwife in the perinatal mental health team made to put her at ease before the birth, including a ‘tour’ of the theatre and suggestions how to make the C-section a positive experience, such as how to facilitate skin-to-skin with the dad immediately after birth. Mandy ended up having a severe haemorrhage during the operation and needed blood transfusions. Nevertheless, she feels at peace with her second birth, because she felt supported and included, and views the complications as unavoidable, and therefore not traumatic: ‘unexpected things happen and that is fine’.

## Discussion

In the following, I offer a critical analysis of the themes presented above by drawing on international literature on informed decision-making and by contextualising the results in the wider context of the Irish maternity system. The aim of this analysis is to move beyond describing *what* is happening, to offering explanations *why* this is happening and *how* women’s subjective experiences are shaped by organisational cultures and social norms.

### The Language of Authority: Telling, not Asking

The narratives presented above demonstrate how medical authority is established through selective use of information (‘what’), and through the paternalistic and authoritative ways of relaying the information (“how”). The set of options offered to women is often severely limited. Information is abbreviated, leaving out side effects and knock-on effects of ‘standard’ procedures such as pain medication and inductions. Thereby, women end up ‘agreeing’ to the option preferred by the health care provider, as Rachel’s comment exemplifies: ‘*I don’t think I understood that I would be put on an oxytocin drip and I didn’t really know what to expect*’. Arguably, these examples constitute failures to offer ‘sufficient information in a manner that is comprehensible to the service user’, as per HSE Consent Policy (HSE, 2019, p. 23). However, this discrepancy between policy guidelines and actual practice is neither surprising nor unusual: it is a common feature of paternalistic health care systems to ‘tailor information to ensure the selection of what the health care expert considers the best choice’ (McLeod & Sherwin, 2000, 267).

Rarely were women presented with various different courses of actions – or indeed, the important option to simply wait and ‘do nothing’ (other than quietly support the physiological process of labour, cf. Huschke, 2021). This is not surprising considering that maternity care providers are bound to the limits of a medicalised model of care, and socialised into the risk-focused approach of this model (cf. Healy et al., 2017). However, this practice limits decision-making, at best, to (vaguely informed) consent – as opposed to informed choice. Consent refers to accepting or refusing a certain course of action. Informed choice entails a wider range of options that take the woman’s social context into account (Kirkham, 2004, p. 278). While the language used in policy documents such as the Irish Maternity Strategy (Department of Health, 2016) has changed, co-opting the rhetoric of choice, practice has not caught up with that yet, overall, as the narratives shared by women in this study indicate.

Importantly, it matters when and how women are asked to consent to a certain procedure. Rachel’s experience is a prime example for ‘consent’ that is given under problematic circumstances that do not facilitate informed decision-making: her consultant suggested to do a membrane sweep *while* he was performing a routine vaginal exam, without any previous discussion of the risks and benefits involved in the induction of labour through a sweep, nor the alternatives. Unfortunately, Rachel’s experience is far from unique: membrane sweeps and artificial rupture of membranes (ARM) are suggested to women in similarly disempowered situations, when clearly, asking questions, gathering information, and weighing options is not ‘possible or practical while one is in the middle of being intimately examined’ (Wickham, 2018, p.100).

In addition, it is apparent that more often than not, decision-making involves women *being told* what to do, or what is going to happen *to* them: ‘*They wanted me to push and they started using the suction cup*’, recalls Sabina, and Alice was informed she could start pushing ‘*at 12 o’clock*’. These experiences mirror the findings of a previous Irish study that reported that ‘women felt spoken at, rather than communicated with’ (O’Hare & Fallon, 2011, p. 167). Instead of adopting an ethic of care these decision-making processes are permeated by an *ethic of control*, where healthcare professionals exercise *power over* the birthing person (cf. Heckert & Clemison, 2011, p. 4).

This authoritative approach to decision-making becomes most apparent when women attempt to refuse the proposed course of action, as Mel’s and Delia’s stories demonstrate. Delia was confronted with the ultimate threat, the ‘dead-baby-card’ (Morton et al., 2018; cf. Jenkinson et al., 2017, p. 6), when she was told by her consultant that her baby ‘*was going to be born dead*’ if she refused induction for her ‘post-dates’ pregnancy. The rhetoric of risk and the practice of invoking fear by predicting negative outcomes is a standard feature of medicalised approaches to birth, observed across national and cultural contexts: ‘physicians often present risk information in such a way that arouses women’s fears, thereby ensuring patient compliance’ (Thachuk, 2007, p. 47; cf. Hall et al., 2012). Confronted with these powerful predictions of harm, women are more likely to hand over their decision-making power to the medical professionals, trusting that ‘they know best’ (cf. Edwards, 2004, p. 20; Montes Muñoz, 2007).

### Hospital Culture: Whom Does it Serve?

Many of the women who participated in this study expressed that they entered the maternity care system with the hope that suggestions made by medical staff are coherently grounded in evidence-based best practice and aim to always serve the wellbeing of the mother and baby. ‘*I kind of trusted him*’, said Rachel about the obstetrician who then proceeded to perform a sweep without her informed consent, and Mandy recalled ‘*trusting that they had my baby and me they had our best interest at heart*’. Both of these women ended up feeling very differently about their relationship with maternity care providers after their negative birth experience: ‘*I will never let anybody go near me again you know*’, explained Rachel, and Mandy felt she had been ‘*treated like an animal who was an inconvenience*’. In the reality of highly medicalized and paternalistic, yet under-resourced maternity systems, there are other factors that influence what is *done to* women, including economic considerations and medical professionals’ personal beliefs and values (Sadler et al., 2016, p. 48, cf. Edwards, 2004, p. 13; Jenkinson et al., 2017, p. 6; Rodríguez-Garrido & Goberna-Tricas, 2021).

In the Irish context, current hospital practice continues to be influenced by one of the prime examples of technocratic approaches to labour and birth, which originated in the Irish National Maternity Hospital: the ‘active management of labour approach’ (AML) developed by Irish obstetricians in the 1970s. In a nutshell, AML aims to ensure that all women give birth within 12 hours upon arrival in the hospital in order to increase the hospital’s cost-efficiency and avoiding staff shortages, mainly by accelerating labour via artificial rupture of membranes or an oxytocin drip (O’Driscoll & Meagher, 1986). The AML approach has been interpreted and modified differently throughout the English-speaking world (e.g. Maffi, 2016) and across Ireland over the past decades. It continues to inform Irish maternity care, as exemplified by the fact the National Maternity Hospital in Dublin still runs AML courses catering to an international obstetric audience.

While the procedure is not followed strictly any longer, the underlying ideological foundations continue to shape how labour and birth are ‘managed’: Firstly, most of the women described a highly interventionist approach to labour and birth and medical providers’ discomfort with and indeed, disapproval of hands-off (i.e. ‘passive’) attitudes to pregnancy and birth, thereby limiting the options made available to women. Secondly, as discussed in the previous section, women felt that they were expected to go along with the protocol, and that they were perceived as unruly troublemakers if they dared to exercise their own judgments throughout pregnancy and labour. Thirdly, some women felt that the management of the hospital’s (limited) resources took precedence over the wellbeing and subjective needs of the birthing person.

In addition to the active management of labour as an underlying philosophy, another structural constraint has become increasingly dominant over the past decade, in Ireland as elsewhere in the world: the culture of litigation. Delia’s story clearly exemplifies how the fear of litigation influences decision-making. Medical litigation is common in Ireland, with pay-outs having increased by 365% between 2014 and 2018 – and around half of all cases relate to maternity care and birth (Finn, 2019). This has severe consequences for the decision-making process during labour and birth: a study in the UK found that ‘fear of litigation, rather than security with evidence-based information, was also reported to be a major factor influencing the ways in which choices were presented to, or withheld from, service users’ (Kirkham & Stapleton, 2004, p. 121).

Lastly, it is important to highlight that the technocratic model of ‘managing’ birth does not only harm women, it also renders it difficult for healthcare providers to offer woman-centred care: ‘women and midwives have been in an impossible situation where the midwife’s need to remain within her policies are pitted against women’s autonomy’ (Edwards, 2004, p. 23, cf. Mander & Melender, 2009; Murphy-Lawless, 2015; Kirkham & Stapleton, 2004, p. 123).

In sum, the findings presented here illustrate the ways in which Irish maternity hospitals – as examples of interventionist, standardised, risk-focused maternity systems – render it difficult for care providers to offer woman-centred care and support informed decision-making (cf. Anderson, 2004, 259). As a last resort, some women – like Zoey – feel that opting out of the system entirely by free birthing their babies is the only way they can make autonomous decisions regarding their births (cf. Dahlen et al., 2011).

### A Healthy Baby is all that Matters: Low Expectations, Omnipresent Fears

In the face of ever-present medical authority, enforced in those countless situations where women are told what to do rather than asked what they want, and a technocratic maternity system that favours standardised procedures over subjective needs and desires, it is, arguably, not easy for women to ‘simply say no’ (Edwards, 2004). Most of the women who took part in this study did not dare to openly disagree with the unilateral decisions made by health professionals on their behalf. Doubts about the decision-making process often only surfaced after the fact, when they reflected on how they had been treated, and what the birth felt like to them – and when they accessed sources of evidence-based information that contradict what they had been told by their care providers. Furthermore, many of the birth stories that women shared unveiled that they do not *expect* birth to be a positive experience, nor do they expect to have any significant role in the decision-making process.

Not surprisingly, all of the women who found the courage to challenge standard procedures and attempted to actively participate in the decision-making process were privileged: educated, white middle class women (both Irish and non-Irish). None of the women in this study who were marginalised – that is, women from a working class background, women of colour, women with a precarious immigration status, and/or women who had experienced State violence (e.g. had their children forcibly taken into care) – described their birth experiences as positive or empowering. None of them dared to openly refuse the interventions that they were subjected to, even if they disapproved of them. The more privileged women in this study had access to a wider range of knowledge aligned with their subjective values (such as books, websites, and/or private obstetricians, midwives and doulas), and the economic means which allowed them to choose their care provider, aligned with their own birth philosophy.

Importantly, however, not all privileged women who were interviewed in this study actively participated in the decision-making process. Several middle class, educated white Irish women described their interactions with care providers in terms of passive obedience, and did not express expectations to be actively involved in the decision-making process, nor did they aim for birth to be a positive or empowering experience. Previous studies have found that socially disadvantaged women are less likely to take active part in the decision-making process than more privileged women (Ebert et al., 2014). The stories shared by the participants in this study indicate that the passive acceptance of disempowering approaches to decision-making may indeed be widespread among economically and socially privileged women (i.e. white middle class Irish women) as well as women who are marginalised.

The omnipresent discourse around birth as dangerous, painful and difficult influences what women believe themselves capable of: women are socialised to believe that birth is risky and needs to be managed by a medical professional (Jomeen, 2006; Ebert et al., 2014; Stoll & Hall, 2013). As a result, birthing people may feel that a healthy baby is the best one can hope for (cf. Hill, 2019, p. 23), and will simply not feel prepared to take the weight of responsibility for their baby’s well-being. This is amplified by the paternalistic, technocratic culture of the Irish maternity system, which it severely limits the options for a non-medicalised physiological birth. It thereby becomes a self-fulfilling prophecy: if the birth stories that circulate are predominantly hospital-based births involving interventions, pain and fear, then it is not surprising that women have difficulty trusting their bodies to birth their babies in their own way. Self-trust, however, is a key prerequisite to exercise autonomy, as McLeod and Sherwin (2000) remind us: ‘without trust in [her own] judgments and trust overall in her ability to exercise choice effectively, any agent would have little motivation to deliberate on alternative courses of action’ (McLeod & Sherwin, 2000, pp. 262–263).

## Implications for Practice: Towards Informed Choice and Person-Centered Care

Some of the women who participated in this study experienced the interactions with care providers during pregnancy and birth as very positive, and felt heard, supported and empowered to give birth in the way that was right for them. Overall, however, this research found that the interactions with maternity care providers rarely promoted women’s empowerment or nourished their autonomy. The ethic of control that permeates the technocratic, medicalised Irish maternity system left some women feeling deeply disappointed, disempowered, unsupported, disrespected, anxious, angry or traumatised. Many of the women expressed that they never expected to have a say in what happens to them, their bodies and their babies during birth. For them, all that mattered was a healthy baby – thereby expressing the social norm that ‘the woman, her feelings, her experience, and her postnatal mental health’ do not matter (Hill, 2019, p. 23). Clearly, this is not good enough, if the aim is to create women-centred services that facilitate choice and recognise pregnancy and birth as ‘major life-changing events’, as expressed in Ireland’s National Maternity Strategy (Department of Health, 2016) and international best practice guidelines (WHO, 2018). To address the shortcomings illustrated by the narratives in this article, I propose two ways of moving forward: structural changes and (inter)personal changes.

The structural changes required include, firstly, a continuity of carer model. International best practice guidelines recommend that pregnant people should have the opportunity to develop a relationship with their care provider, as this can enhance their sense of autonomy and significantly increase their involvement in decision-making, and consequently, lead to a more positive birth (WHO, 2018, cf. Holten et al., 2018). Secondly, more options regarding the care provider and the place of birth are needed, including an expansion of homebirth services and the establishment of stand-alone midwifery-led units. Thirdly, time and staff resources are required to find out about the needs and desires of the birthing person and talk through options (cf. Thachuk, 2007, p. 51).

Changing the maternity system in a top-down approach to offer women a wider range of better options is necessary, but it is not enough. Autonomy is co-created by women who have the chance to develop self-trust through reflecting on their needs and views, acting on them, and experiencing what it feels like to trust her own judgment (cf. McLeod & Sherwin, 2000) – and health care providers who are *willing* to facilitate autonomous decision-making, and have the skills and resources to do this work (cf. Dove et al., 2017). The inner work needed to form professional relationships that respect what people desire (cf. Delgado, 2019) would involve *un*learning the hierarchical, authoritarian and often patronising ways of engaging with birthing people that often become habitual and unconscious when working in technocratic maternity systems.

At the core of this work lies self-reflexivity, which involves awareness and transparency regarding one’s own social position, values and beliefs and how they shape what care providers say to birthing people and how they say it, the suggestions they make, the choices they prefer, the options they omit (cf. Jenkinson et al., 2017). Unequal power dynamics are omnipresent in technocratic maternity settings, and it is not enough to simply state that the birthing person has the right to make their own decisions: the care provider needs to relinquish power, while at the same time finding ways of nourishing the birthing person’s self-trust as a requirement for autonomous decision-making (cf. Briceño Morales et al., 2018).

With these changes, health care professionals, particularly midwives, would find it easier to offer truly person-centred care, while at the same time inspiring women to reclaim birth as a deeply personal, transformative and empowering experience.

## Uncited References

Dove et al., 2017.

## Notes

1. All participants identified as *women*. However, I am using the terms *pregnant people* and *birthing person* interchangeably with the terms *pregnant women/mother* to be inclusive of the experiences of pregnant trans and nonbinary people in the wider population – that is, people who do not identify as cis-gender women.

2. All participants were given the opportunity to read the article before publication and approved the use of pseudonyms.
